# Age of Information of Parallel Server Systems with Energy Harvesting

**DOI:** 10.3390/e23111549

**Published:** 2021-11-21

**Authors:** Josu Doncel

**Affiliations:** Mathematics Department, University of the Basque Country, UPV/EHU, 48940 Leioa, Spain; josu.doncel@ehu.eus

**Keywords:** parallel servers, energy harvesting, Age of Information

## Abstract

Motivated by current communication networks in which users can choose different transmission channels to operate and also by the recent growth of renewable energy sources, we study the average Age of Information of a status update system that is formed by two parallel homogeneous servers and such that there is an energy source that feeds the system following a random process. An update, after getting service, is delivered to the monitor if there is energy in a battery. However, if the battery is empty, the status update is lost. We allow preemption of updates in service and we assume Poisson generation times of status updates and exponential service times. We show that the average Age of Information can be characterized by solving a system with eight linear equations. Then, we show that, when the arrival rate to both servers is large, the average Age of Information is one divided by the sum of the service rates of the servers. We also perform a numerical analysis to compare the performance of our model with that of a single server with energy harvesting and to study in detail the aforementioned convergence result.

## 1. Introduction

### 1.1. Motivation

The Age of Information is a recent metric of the performance of systems and it measures the freshness of the information that a monitor has about the status of a remote process of interest. There is a wide range of applications in which information about a source must be as recent as possible. An example of this is given in autonomous driving systems since the location of the vehicles must be known as soon as possible. Or, in other words, obsolete information about the traffic might lead to bad consequences (traffic accidents, for instance) to the users.

Status update systems are formed by sources of generation status updates, a transmission channel and a monitor that receives the updates. The transmission channel takes care of sending the status updates from the source to the destination. It is clear that the devices of the transmission channel require energy to work. Therefore, it is important to consider energy consumption in the modeling of the transmission channel. Furthermore, there has been recently an increasing amount of different types of renewable energy sources that feed the energy network. Some examples are solar or wind energy sources, which are clearly very volatile. As a consequence, the randomness of the generation of energy also needs to be taken into account in the modeling of the transmission channel.

Current communication networks are very complex and often allow users to operate using different transmission channels. This is the case, for instance, when a user is a part of an overlay network (i.e., when it belongs to a set of nodes that are located in different spots over the Internet and collaborate with each other to forward data between any pair of nodes with minimum delay). In fact, in this instance, the user can choose the transmission channel that provides the IP protocol or through the overlay network. Therefore, in this work, we study the average Age of Information in a status update system with energy harvesting. That is, we consider that the transmission channel is formed by parallel servers that do not interchange information and a battery that can store energy that can be used to send status updates after getting service in the servers.

### 1.2. Related Work

The Age of Information has been introduced in [1,2] as a metric to measure the freshness of the information about the state of a remote system. Since its introduction, there has been many researcher of different areas that has been interested in analyzing this metric. In the first works following the seminal papers, the goal has been to characterize the average Age of Information of status update systems where the transmission channel is modeled as a single queue. For instance, the authors in [3] characterize the average Age of Information of a single server (i.e., a queue without buffer) and a single source. Regarding optimality, the authors in [4] show that the preemptive Last Generated First Served policy minimizes the Age of Information. Unfortunately, the characterization of the average Age of Information of many models is known to be an extremely difficult task. Therefore, some authors has been interested in other similar metric of performance such as the Peak Age of Information [5] or the Age of Incorrect Information [6]. We refer to the following surveys on this topic for full details of these metrics and their properties [7,8,9].

Let us now discuss the work of some authors that have been interested in analyzing the Age of Information of a system with energy harvesting. In [10,11] it is considered a system with Poisson arrivals of energy and that there is no losses of packets. Their goal is to find the optimal status updates policy such that the battery is not empty upon an arrival of a status update. The authors in [12,13,14] generalize the model of [10,11] by allowing status update losses and also focus on optimal policies for generation updates, with or without knowledge (or feedback) whether the status updates are delivered successfully. Our model, that has been in inspired by the Energy Packet Networks [15,16], is different from these models for different reasons. First, we do not impose the presence of energy to receive a status update. Another difference is that the generation of status updates follows a Poisson process in our model, which is not the case in these works. Finally, our goal is different since we are interested in characterizing the average Age of Information and studying its properties and, hence, we do not aim to find the optimal policy.

### 1.3. Contribution

We consider a system with two parallel homogeneous servers and one battery that stores energy packets. Energy packets model a certain amount of energy and are necessary to send the status updates (or data packets) to the monitor after ending service. This means that a data packet is sent to the battery when it ends service and, if the battery is empty, the data packet is lost, whereas if battery is not empty the data packet is delivered to the monitor and one energy packet disappears. We consider that arrivals of data packets and energy packets follow a Poisson process and the queues that handle data packets and energy packets do not have buffer. We allow preemption of data packets, i.e., when a data packet arrives to a server that is busy, the incoming packet replaces the packet in service.

The first contribution of this work is to characterize the average Age of Information of the above status update system using the Stochastic Hybrid System technique [17]. More specifically, we show that the average Age of Information can be computed by solving a system of 8 linear equations. We then consider the regime where the arrival rate of data packets to both servers tends to infinity and we show that the average Age of Information is one divided by two times the service rate of data packets.

The model we study here generalizes

- the work of Section IIIA of [18] where it is studied the Age of Information of two parallel servers. In our work, we consider energy harvesting in their model. In fact, when in our model the arrival rate of energy packets is very large, it coincides with the model of [18].
- the work of [19] where it is analyzed a system with a single server and energy harvesting. In our work, we consider the same energy harvesting model, but with two parallel servers.

We go beyond the above presented analytical results with a numerical work that we describe next. First, we aim to compare the performance of a single servers with two parallel servers with energy harvesting. For this purpose, we consider the following systems: (i) a single server with arrival rate λ/2 and service rate μ, (ii) a single server with arrival rate λ and service rate 2μ and (iii) two parallel servers with service rate μ, each of them handling an arrival rate of λ/2. Let us note that the ratio of the arrival rate over the service rate coincide in all the servers of the systems under consideration. This comparison has been previously done in Section IIIA of [18], but they do not consider energy harvesting. Our first finding is that, when the arrival rate of energy packets is very large, we obtain the plot as Figure 4 of [18] and, therefore, their conclusions follow in our model as well (i.e., the system with double service rate and a single server minimizes the average Age of Information). We then investigate whether the conclusions of [18] also hold when the arrival rate of energy packets is not large. We observe that the average Age of Information is smaller for the system with two parallel servers and this difference increases when we decrease the arrival rate of energy packets. Finally, we study how the average Age of Information converges, when the arrival rate to the servers increases, to the value obtained in our analytical part. We conclude that the average Age of Information is not monotone with respect to the arrival rate of energy packets when the arrival rate to both servers is small. However, the average Age of Information does not depend on the arrival rate of energy packets when the arrival rate of packets to both servers is very large.

Potential applications of this model include systems in which two different transmission channels can be chosen to send updates. This is the case, for instance, when the source that generates status updates is part of an overlay network (i.e., when it belongs to a set of nodes that are located in different spots over the Internet and collaborate with each other to forward data between any pair of nodes with minimum delay) and it can choose to send the status updates through the path the provides the IP protocol or through the overlay routing.

### 1.4. Organization

The rest of the paper is organized as follows. First, in Section 2, we describe the model we study in this article. The average Age of Information analysis of this model is presented in Section 3. In Section 4, we focus on our numerical work and, finally, in Section 5, we draw the main conclusions of this work.

## 2. Model Description

### 2.1. Age of Information

We study the transmission of status updates (or data packets) to a monitor. We consider that data packet *i* is generated at time $t_{i}$ and that it is delivered to the monitor at time $t_{i}^{\prime }$. We denote by N(t) the index of the last successfully delivered data packet to the monitor at time *t*, i.e.,

$$N\left(t\right)=\max\left\{i|t_{i}^{\prime }\leq t\right\}.$$

**Figure 1:**
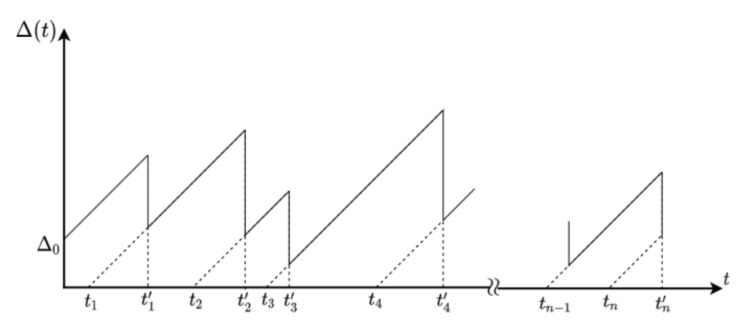
An example of Δ(t).

Taking into account that the generation time of the last received data packet before time *t* is $t_{N(t)}$, we define the Age of Information at time *t* as follows:

$$\Delta \left(t\right)=t-t_{N(t)},$$

that is, the Age of Information at time *t* is the time elapsed since the generation of the last delivered packet to the monitor. We show in Figure 1 an example of Δ(t).

Assuming that the updating system is stable, the average Age of Information can be computed as the area below a “saw-tooth” shaped curve with teeth at the times at which the data packets are delivered (see Figure 1). Hence, if we denote by Δ the average Age of Information, we have that

$$\Delta =\lim_{\tau \to \infty }\frac{1}{\tau }\int_{0}^{\tau }\Delta \left(t\right)dt.$$

In this article, we are interested in calculating the average Age of Information in an energy harvesting model. In the following section, we describe the model we analyze.

### 2.2. Energy Harvesting Model

**Figure 2:**
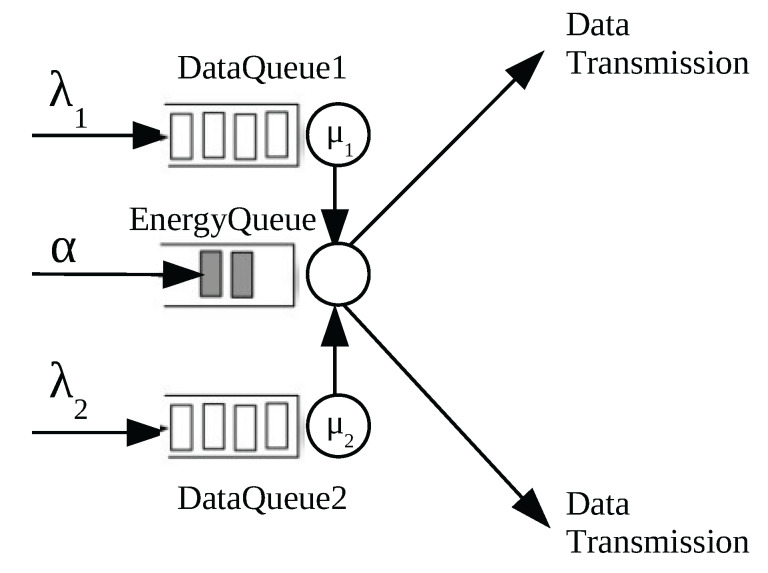
The energy harvesting model with two parallel data queues and a single energy queue. Energy packets are depicted with gray and data packets with white.

In our model, we represent energy by packets of discrete units called energy packets that model a certain quantity of energy (energy packets) measured in Joules, whereas the status updates of a process of interest are represented by packets that we call data packets. We consider an energy harvesting model formed by two parallel queues that store data packets (data queues) and a single queue that stores the energy packets. We show in Figure 2 the model under consideration in this work.

Energy packets arrive to the system according to a Poisson process of rate α and data packets (or workload packets) with rate λ. Upon arrival, a packet is dispatched to data queue 1 with probability p>0 and to data queue 2 with probability 1−p>0. Therefore, the arrival rate to data queue 1 is $\lambda_{1}=\lambda p$ and to data queue 2 is $\lambda_{2}=\lambda \left(1-p\right)$.

**Remark** **1.** *The probability p can be seen as the willingness of a source to use an alternative path (for instance, the path of an overlay network) rather than the usual transmission channel.*

We consider that the service rate of jobs of data queue *i* is exponentially distributed with rate μ, i = 1, 2.

In this model, we consider that data packets, i.e., the packets of the data queues, start the transfer to a single energy queue. This means that, when a data packet gets served (in data queue 1 or data queue 2), it is sent to the energy queue. If the energy queue is empty upon arrival of a data packet, the data packet is lost. However, if there are energy packets when a data packet arrives to the energy queue, the data packet is transferred successfully to the monitor and one energy packet disappears.

**Remark** **2.** *Our model considers a single energy queue. This models that the destination requires energy to receive status updates. This occurs, for instance, when there is a wireless antenna in charge of receiving the status updates at the destination (indeed, in absence of energy the antenna cannot deliver packets to the monitor).*

Here, we assume that the energy queue and the data queues do not have buffer. Therefore, the number of packets in each queue is, at most, one. Besides, energy packets that arrive when the energy queue is full are dropped, whereas when a data packet arrives to a full data queue, it replaces the job in execution.

## 3. Average Age of Information Analysis

**Figure A1:**
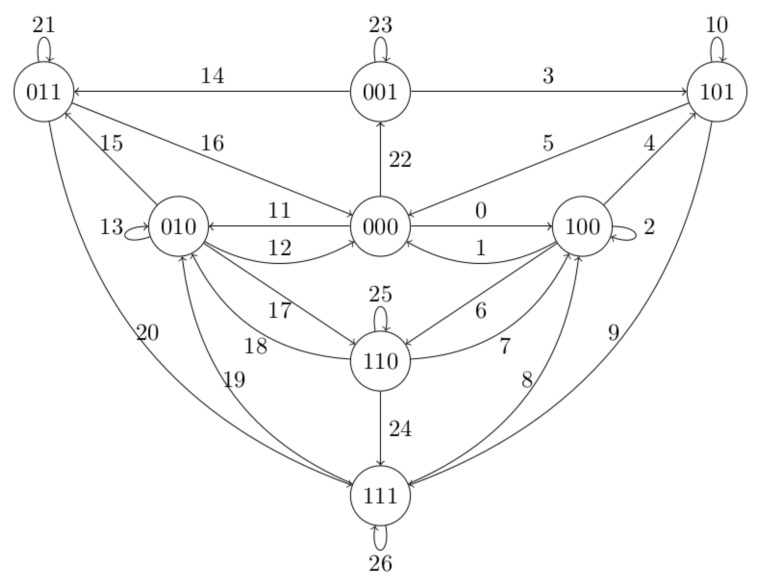
The SHS Markov chain for the model with two parallel data queues and a battery.

In this section, we aim to analyze the average Age of Information of a system formed by two parallel queues with energy harvesting. We will use the Stochastic Hybrid System method to characterize the average Age of Information of the system under consideration. The Stochastic Hybrid System is formed by two values: the state of a continuous time Markov Chain and a vector containing the generation times of all the packets in the system as well of the current Age of Information. The Markov chain we consider is presented in Figure A1.

Let $s_{0}\;s_{1}\ldots \;s_{7}$ be the solution of the following system of equations:

(1a) $$\begin{array}{cc} 0 & =-s_{0}\left(\lambda +\alpha \right)+s_{2}\mu +s_{3}\mu +s_{4}\mu +s_{5}\mu \end{array}$$

(1b) $$\begin{array}{cc} 0 & =s_{0}\alpha -s_{1}\lambda \end{array}$$

(1c) $$\begin{array}{cc} 0 & =s_{0}\lambda \left(1-p\right)-s_{2}\left(\lambda p+\mu +\alpha \right)+s_{6}\mu +s_{7}\mu \end{array}$$

(1d) $$\begin{array}{cc} 0 & =s_{1}\lambda \left(1-p\right)+s_{2}\alpha -s_{3}\left(\lambda p+\mu \right) \end{array}$$

(1e) $$\begin{array}{cc} 0 & =s_{0}\lambda p-s_{4}\left(\lambda \left(1-p\right)+\alpha +\mu \right)+s_{6}\mu +s_{7}\mu \end{array}$$

(1f) $$\begin{array}{cc} 0 & =s_{1}\lambda p+s_{4}\alpha -s_{5}\left(\lambda \left(1-p\right)+\mu \right) \end{array}$$

(1g) $$\begin{array}{cc} 0 & =s_{2}\lambda p+s_{4}\lambda \left(1-p\right)-s_{6}\left(\alpha +2\mu \right) \end{array}$$

(1h) $$\begin{array}{cc} 0 & =s_{3}\lambda p+s_{5}\lambda \left(1-p\right)+s_{6}\alpha -s_{7}\;2\mu \end{array}$$

that satisfies that $\sum_{i=0}^{7}s_{i}=1$. As we will see in Appendix B, the solution of the above system of equations provides the steady-state distribution of the Markov chain of Figure A1.

We also define $x_{1},x_{2},\ldots ,x_{16}$ as the solution of the following system of equations:

(2a) $$\begin{array}{cc} -s_{0}= & -x_{1}\left(\lambda +\alpha \right)+\mu x_{7}+\mu x_{10}+\mu x_{3}+\mu x_{6} \end{array}$$

(2b) $$\begin{array}{cc} -s_{1}= & -x_{2}\left(\lambda +\alpha \right)+\alpha x_{1}+\alpha x_{2} \end{array}$$

(2c) $$\begin{array}{cc} -s_{2}= & -x_{3}\left(\lambda +\alpha +\mu \right)+\lambda \left(1-p\right)x_{1}+\lambda \left(1-p\right)x_{3}\\ & +\mu x_{15} \end{array}$$

(2d) $$\begin{array}{cc} -s_{2}= & -x_{4}\left(\lambda +\alpha +\mu \right)+\mu x_{16} \end{array}$$

(2e) $$\begin{array}{cc} -s_{3}= & -x_{5}\left(\lambda +\alpha +\mu \right)+\alpha x_{3}+\alpha x_{5}+\lambda \left(1-p\right)x_{2}\\ & +\lambda \left(1-p\right)x_{5} \end{array}$$

(2f) $$\begin{array}{cc} -s_{3}= & -x_{6}\left(\lambda +\alpha +\mu \right)+\alpha x_{4}+\alpha x_{6} \end{array}$$

(2g) $$\begin{array}{cc} -s_{4}= & -x_{7}\left(\lambda +\alpha +\mu \right)+\lambda px_{1}+\lambda px_{7}+\mu x_{16} \end{array}$$

(2h) $$\begin{array}{cc} -s_{4}= & -x_{8}\left(\lambda +\alpha +\mu \right)+\mu x_{15} \end{array}$$

(2i) $$\begin{array}{cc} -s_{5}= & -x_{9}\left(\lambda +\alpha +\mu \right)+\lambda px_{2}+\lambda px_{9}+\alpha x_{9}+\alpha x_{7} \end{array}$$

(2j) $$\begin{array}{cc} -s_{5}= & -x_{10}\left(\lambda +\alpha +\mu \right)+\alpha x_{10}+\alpha x_{8} \end{array}$$

(2k) $$\begin{array}{cc} -s_{6}= & -x_{11}\left(\lambda +2\mu +\alpha \right)+\lambda px_{3}+\lambda px_{11}\\ & +\lambda \left(1-p\right)x_{7}+\lambda \left(1-p\right)x_{11} \end{array}$$

(2l) $$\begin{array}{cc} -s_{6}= & -x_{12}\left(\lambda +2\mu +\alpha \right)+\lambda \left(1-p\right)x_{8}+\lambda \left(1-p\right)x_{12} \end{array}$$

(2m) $$\begin{array}{cc} -s_{6}= & -x_{13}\left(\lambda +2\mu +\alpha \right)+\lambda px_{4}+\lambda px_{13} \end{array}$$

(2n) $$\begin{array}{cc} -s_{7}= & -x_{14}\left(\lambda +2\mu \right)+\lambda px_{5}+\lambda px_{14}+\lambda \left(1-p\right)x_{9}\\ & +\lambda \left(1-p\right)x_{14}+\alpha x_{11} \end{array}$$

(2o) $$\begin{array}{cc} -s_{7}= & -x_{15}\left(\lambda +2\mu \right)+\lambda \left(1-p\right)x_{10}+\lambda \left(1-p\right)x_{15}\\ & +\alpha x_{12} \end{array}$$

(2p) $$\begin{array}{cc} -s_{7}= & -x_{16}\left(\lambda +2\mu \right)+\lambda px_{6}+\lambda px_{16}+\alpha x_{13}, \end{array}$$

where $s_{0},s_{1},\ldots ,s_{7}$ are given in Equation (1a–h). As we explain in Appendix B, the values $x_{1},\ldots ,x_{16}$ coincide with the generation time of all the packets in the system for all the possible states of the Markov chain.

In the following result, we use the Stochastic Hybrid System technique [17] to characterize the average Age of Information of this system and we show that it can be done by solving the above system of equations.

**Proposition** **1.** *The average Age of Information of a system with two parallel servers with the energy harvesting is given by*

$$x_{1}+x_{2}+x_{3}+x_{5}+x_{7}+x_{9}+x_{11}+x_{14},$$

*where*$x_{1},x_{2},\ldots ,x_{16}$*are the solution of Equation* (2a–p)*.*

**Proof.**  See Appendix B. □

In Proposition 1, we show that the computation of the average Age of Information of the system under study requires to solve Equation (2a–p), which is a system of 16 linear equations with 16 variables. Now, we aim to show that this system of equations has a special structure and how it can be used to obtain a method to compute the average Age of Information by solving a simpler system. Let us first present the following auxiliary results.

**Lemma** **1.** *The Equation* (2d,f,m,p), *form a system of 4 linear equations with 4 variables (*$x_{4},x_{6},x_{13}$ *and* $x_{16}$*). Let*

(3) $$c=\frac{\lambda p\alpha }{\lambda +\alpha +\mu }\left(\frac{1}{\lambda +\mu }+\frac{1}{\lambda (1-p)+2\mu +\alpha }\right).$$

*We have that*

(4) $$\begin{array}{cc} x_{16} & =\frac{s_{7}-\frac{\lambda ps_{3}}{\lambda +\mu }-\frac{\alpha s_{6}}{\lambda (1-p)+2\mu +\alpha }-c\;s_{2}}{c\mu -(\lambda (1-p)+2\mu )}, \end{array}$$

*and*

(5) $$\begin{array}{cc} x_{4} & =\frac{\mu x_{16}+s_{2}}{\lambda +\alpha +\mu }. \end{array}$$

*as well as*

(6) $$\begin{array}{cc} x_{6} & =\frac{\alpha x_{4}+s_{3}}{\lambda +\mu }. \end{array}$$

**Proof.**  See Appendix C. □

**Lemma** **2.** *The Equation* (2h,j,l,o)*, form a system of 4 linear equations with 4 variables (*$x_{8},x_{10},x_{12}$ *and* $x_{15}$*). Let*

(7) $$d=\frac{\lambda (1-p)\alpha }{\lambda +\alpha +\mu }\left(\frac{1}{\lambda +\mu }+\frac{1}{\lambda p+2\mu +\alpha }\right).$$

*We have that*

(8) $$\begin{array}{cc} x_{15} & =\frac{s_{7}-\frac{\lambda ps_{5}}{\lambda +\mu }-\frac{\alpha s_{6}}{\lambda (1-p)+2\mu +\alpha }-d\;s_{4}+}{d\mu -(\lambda (1-p)+2\mu )}, \end{array}$$

*and*

(9) $$\begin{array}{cc} x_{8} & =\frac{\mu x_{15}+s_{4}}{\lambda +\alpha +\mu }. \end{array}$$

*as well as*

(10) $$\begin{array}{cc} x_{10} & =\frac{\alpha x_{8}+s_{5}}{\lambda +\mu }. \end{array}$$

**Proof.**  The proof is symmetric to the proof of Lemma 1 and, therefore, we omit it for clarity of the presentation. □

We now write Equation (2a–p) that have not been analyzed in the previous lemmas:

(11a) $$\begin{array}{cc} -s_{0}= & -x_{1}\left(\lambda +\alpha \right)+\mu x_{7}+\mu x_{10}+\mu x_{3}+\mu x_{6} \end{array}$$

(11b) $$\begin{array}{cc} -s_{1}= & -x_{2}\lambda +\alpha x_{1} \end{array}$$

(11c) $$\begin{array}{cc} -s_{2}= & -x_{3}\left(\lambda p+\alpha +\mu \right)+\lambda \left(1-p\right)x_{1}+\mu x_{15} \end{array}$$

(11d) $$\begin{array}{cc} -s_{3}= & -x_{5}\left(\lambda p+\mu \right)+\alpha x_{3}+\lambda \left(1-p\right)x_{2} \end{array}$$

(11e) $$\begin{array}{cc} -s_{4}= & -x_{7}\left(\lambda \left(1-p\right)+\alpha +\mu \right)+\lambda px_{1}+\mu x_{16} \end{array}$$

(11f) $$\begin{array}{cc} -s_{5}= & -x_{9}\left(\lambda \left(1-p\right)+\mu \right)+\lambda px_{2}+\alpha x_{7} \end{array}$$

(11g) $$\begin{array}{cc} -s_{6}= & -x_{11}\left(2\mu +\alpha \right)+\lambda px_{3}+\lambda \left(1-p\right)x_{7} \end{array}$$

(11h) $$\begin{array}{cc} -s_{7}= & -x_{14}2\mu +\lambda px_{5}+\lambda \left(1-p\right)x_{9}+\alpha x_{11} \end{array}$$

which is a system of 8 equations with 12 variables (the variables are $x_{1},x_{2},x_{3},x_{5},x_{6},x_{7},x_{8},x_{9},$$x_{10},x_{11},x_{14},x_{15}$ and $x_{16}$). However, an explicit expression of $x_{6}$ and $x_{16}$ have been obtained in Lemma 1 and an explicit explicit expression of $x_{10}$ and $x_{15}$ in Lemma 2. Therefore, the next result follows.

**Proposition** **2.** *Let*$x_{1},x_{2},x_{3},x_{5},x_{7},x_{9},x_{11},x_{14}$*be the solution of Equation* (11a–h) *(recall that* $x_{6}$ *and* $x_{16}$ *are given in Lemma 1 and* $x_{10}$ *and* $x_{15}$ *in Lemma 2). Therefore, the average Age of Information of a two parallel servers system with energy harvesting is given by*

$$x_{1}+x_{2}+x_{3}+x_{5}+x_{7}+x_{9}+x_{11}+x_{14}.$$

### 3.1. Analysis When λ Tends to ∞

We now consider the asymptotic regime where λ→∞ when the parameters α and μ are finite.

We first focus on the solution of Equation (1a–h).

**Lemma** **3.** *When*λ→∞*and*max(α,μ)<0*, the solution of Equation* (1a–h) *satisfies that* $s_{0}=s_{1}=s_{2}=s_{3}=s_{4}=s_{5}=0$*.*

**Proof.**  See Appendix D. □

From this result, we conclude that, in the asymptotic regime under study, $s_{6}+s_{7}=1$ and that Equation (11a–h) can be written as

(12a) $$\begin{array}{cc} 0= & -x_{1}\left(\lambda +\alpha \right)+\mu x_{7}+\mu x_{10}+\mu x_{3}+\mu x_{6} \end{array}$$

(12b) $$\begin{array}{cc} 0= & -x_{2}\lambda +\alpha x_{1} \end{array}$$

(12c) $$\begin{array}{cc} 0= & -x_{3}\left(\lambda p+\alpha +\mu \right)+\lambda \left(1-p\right)x_{1}+\mu x_{15} \end{array}$$

(12d) $$\begin{array}{cc} 0= & -x_{5}\left(\lambda p+\mu \right)+\alpha x_{3}+\lambda \left(1-p\right)x_{2} \end{array}$$

(12e) $$\begin{array}{cc} 0= & -x_{7}\left(\lambda \left(1-p\right)+\alpha +\mu \right)+\lambda px_{1}+\mu x_{16} \end{array}$$

(12f) $$\begin{array}{cc} 0= & -x_{9}\left(\lambda \left(1-p\right)+\mu \right)+\lambda px_{2}+\alpha x_{7} \end{array}$$

(12g) $$\begin{array}{cc} -s_{6}= & -x_{11}\left(2\mu +\alpha \right)+\lambda px_{3}+\lambda \left(1-p\right)x_{7} \end{array}$$

(12h) $$\begin{array}{cc} -s_{7}= & -x_{14}2\mu +\lambda px_{5}+\lambda \left(1-p\right)x_{9}+\alpha x_{11} \end{array}$$

If we replace the last equation by the sum the last two equations, we get the following equivalent system:

(13a) $$\begin{array}{cc} 0= & -x_{1}\left(\lambda +\alpha \right)+\mu x_{7}+\mu x_{10}+\mu x_{3}+\mu x_{6} \end{array}$$

(13b) $$\begin{array}{cc} 0= & -x_{2}\lambda +\alpha x_{1} \end{array}$$

(13c) $$\begin{array}{cc} 0= & -x_{3}\left(\lambda p+\alpha +\mu \right)+\lambda \left(1-p\right)x_{1}+\mu x_{15} \end{array}$$

(13d) $$\begin{array}{cc} 0= & -x_{5}\left(\lambda p+\mu \right)+\alpha x_{3}+\lambda \left(1-p\right)x_{2} \end{array}$$

(13e) $$\begin{array}{cc} 0= & -x_{7}\left(\lambda \left(1-p\right)+\alpha +\mu \right)+\lambda px_{1}+\mu x_{16} \end{array}$$

(13f) $$\begin{array}{cc} 0= & -x_{9}\left(\lambda \left(1-p\right)+\mu \right)+\lambda px_{2}+\alpha x_{7} \end{array}$$

(13g) $$\begin{array}{cc} -s_{6}= & -x_{11}\left(2\mu +\alpha \right)+\lambda px_{3}+\lambda \left(1-p\right)x_{7} \end{array}$$

(13h) $$\begin{array}{cc} -1= & -\left(x_{14}+x_{11}\right)2\mu +\lambda px_{5}+\lambda \left(1-p\right)x_{9}\\ & +\lambda px_{3}+\lambda \left(1-p\right)x_{7} \end{array}$$

We now analyze the solution of Equation (13a–h) for large λ.

**Lemma** **4.** *When*λ→∞*and*max(α,μ)<0*, the solution of Equation* (13a–h) *satisfies that* $x_{1}=x_{2}=x_{3}=x_{5}=x_{7}=x_{9}=0$*.*

**Proof.**  The proof uses the same arguments than those of the proof of Lemma 3 and, therefore, we omit it. □

From the above results, we conclude that the average Age of Information of this system is given by $x_{11}+x_{14}$. Furthermore, using that $x_{3}=x_{5}=x_{7}=x_{9}=0$ and from (13h), we obtain that $x_{11}+x_{14}=\frac{1}{2\mu }$, which gives the following result:

**Proposition** **3.** *When* λ→∞ *and* max(α,μ)<∞ *, the average Age of Information of a two parallel servers system with energy harvesting is given by* $\frac{1}{2\mu }$ *.*

It is important to remark that the average Age of Information of the system under study in the considered asymptotic regime does not depend on the arrival rate of energy packets, i.e., on α.

### 3.2. Limitations to Analyze More Complex Models

We have tried to extend the results presented in this section to more complex systems and we have noticed that this task is extremely difficult. The main reason for this that the Markov chain to be considered (and, as a consequence, the number of equations to be solved ) increases at a very high rate with the complexity of the system. This suggests that the analysis of the average Age of Information of more complex systems requires to consider other techniques such as simulations or approximation techniques.

## 4. Performance Evaluation

In the previous section, we have obtained an explicit expression of the average Age of Information of a system with two parallel servers and energy harvesting. Now, we aim to evaluate the obtained expression to analyze its main properties. We have performed a large number of simulations changing the values of the parameters and the illustrations of this section are illustrative of the general pattern. (The plots of this section can be reproduced using the code of https://github.com/josudoncel/AioParallelEnergy, accessed on 18 November 2021).

### 4.1. Parallel Servers vs. Single Server

We aim to compare the average Age of Information of the model under study in this paper with that of a system with a single server with energy harvesting (the average Age of Information of the latter model has been studied in [19]). For this purpose, we consider the following systems: (i) a single server with arrival rates λ and α of data packets and energy packets, respectively, and service rate 2μ (which is represented with a dotted line); (ii) a single server with arrival rates λ/2 and α of data packets and energy packets, respectively, and service rate 2μ (which is represented with a solid line); and (iii) two parallel servers with p=0.5, i.e., the arrival rate to each server is λ/2, the service rate is μ and the arrival rate of energy packets is α (which is represented with a dashed line).

**Figure 3:**
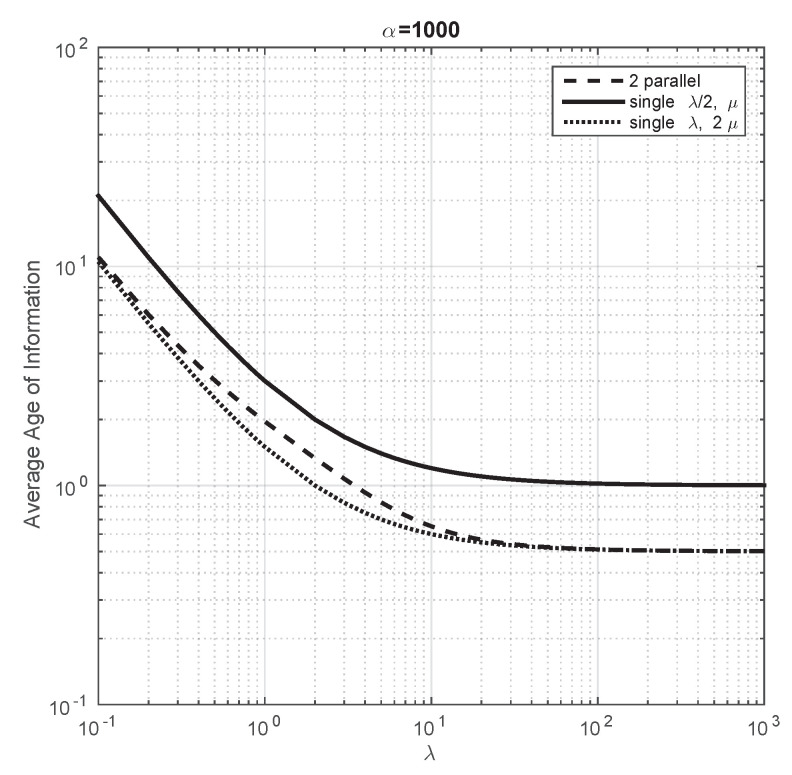
Average Age of Information of the three systems under comparison when λ changes from 0.1 to $10^{3}$. α=1000 and μ=1.

We first consider that the arrival rate of energy packet is very large. For this instance, there is always energy to transmit the data packet when it ends service, or in other words, the data packets are never lost because there is no energy. We note that, when this occurs, the comparison study we carry out here coincides with the analysis of Section III2 of [18]. In Figure 3, we consider μ=1 and $\alpha =10^{3}$ and we plot the evolution of the average Age of Information of the systems under study in this section when λ varies from 0.1 to $10^{3}$. We observe that the obtained plot coincides with Figure 4 of [18]. From this illustration, the authors in [18] conclude that some properties of the classical performance metrics of queuing theory, such as mean delay, are verified for the average Age of Information (the system that minimizes performance is the single server with service 2μ), but other properties do not (the mean delay of a system with two parallel servers with arrival rate to each equal to λ/2 is the same as that of a single server with arrival rate λ/2, but this is not the case for the average Age of Information). This illustration shows that, as expected, these conclusions also hold for our model when α is large.

**Figure 4:**
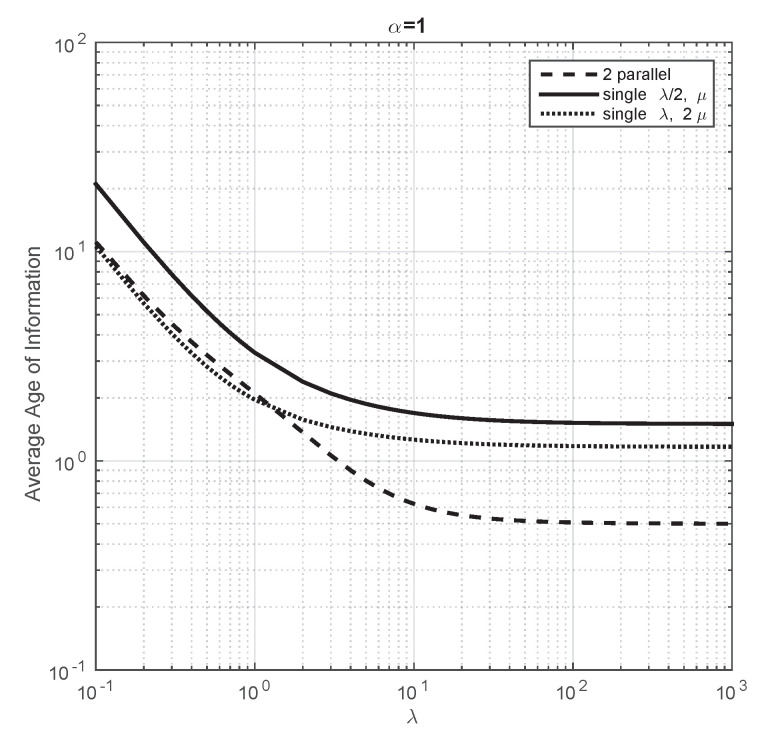
Average Age of Information of the three systems under comparison when λ changes from 0.1 to $10^{3}$. α=1 and μ=1.

**Figure 5:**
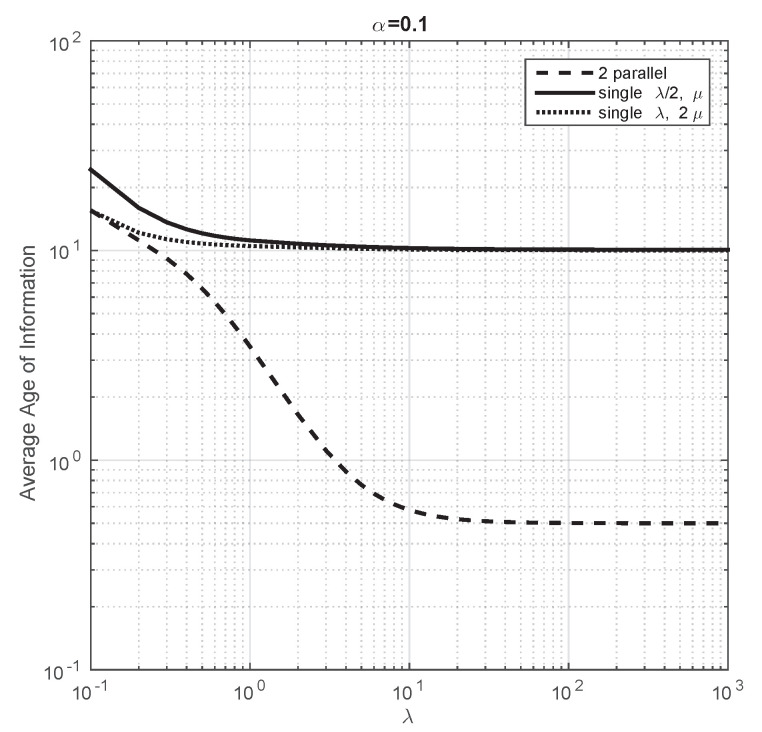
Average Age of Information of the three systems under comparison when λ changes from 0.1 to $10^{3}$. α=0.1 and μ=1.

We now aim to compare the performance of these systems when the arrival rate is not large. Thus, we fix the parameters equal to the previous plot and we consider different values of α. First, we consider α=1 and in Figure 4, we observe that the average Age of Information of all the systems do not change substantially with respect to the previous plot when λ is small. However, as λ grows, the average Age of Information of the systems with a single server decreases less than that of two parallel servers. We have also seen that, when λ is large, the average Age of Information of two parallel servers is 0.5, of a single server with double service rate 1.5 and of a single server with half traffic is 1.67. The difference on the average Age of Information between these system is even larger if we consider a smaller value of α. For instance, in Figure 5, we illustrate that the system with the smallest average Age of Information is the system with two parallel servers for almost all the values of λ we have considered.

### 4.2. Convergence Analysis of Proposition 3

**Figure 6:**
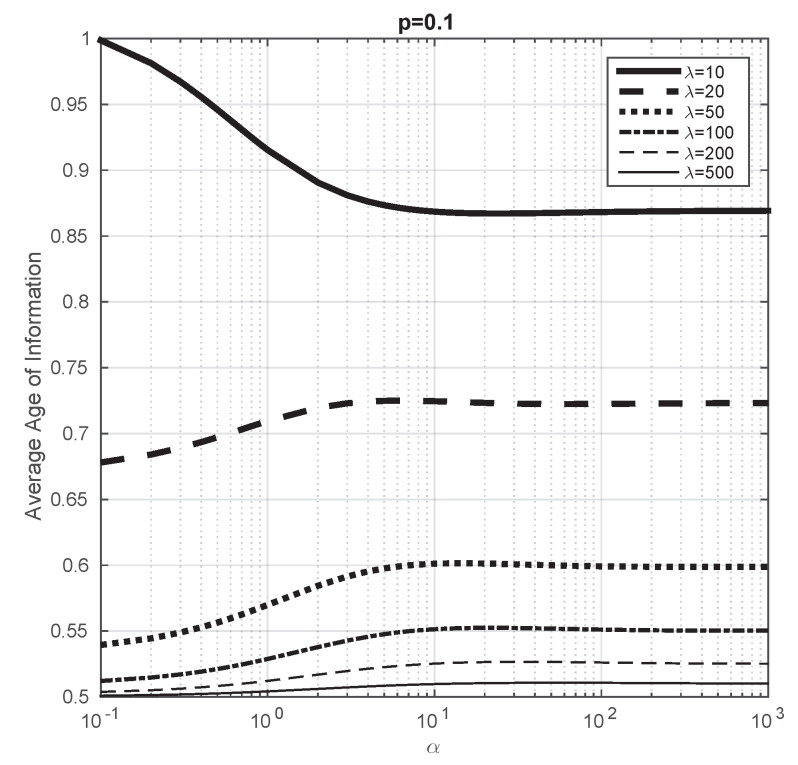
Average Age of Information with respect to α for different values of λ, when α varies from 0.1 to $10^{3}$. μ=1 and p=0.1.

**Figure 7:**
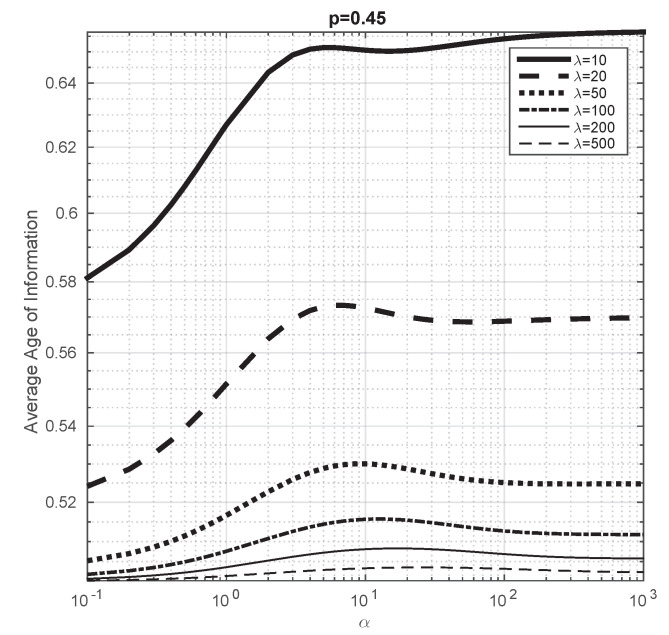
Average Age of Information with respect to α for different values of λ, when α varies from 0.1 to $10^{3}$. μ=1 and p=0.45.

In Proposition 3, we show that, when λ→∞, the average Age of Information tends to $\frac{1}{2\mu }$, i.e., it does not depend on α or *p*. In this part of the article, we aim to study this convergence. We consider μ=1 and p=0.1 and we vary α from 0.1 to $10^{3}$. We plot the evolution of the average Age of Information for different values of λ in Figure 6 and we observe that, as λ increases, the obtained values tend to 0.5, which confirms the result of Proposition 3. We consider p=0.45 in Figure 7 to study how this convergence depends on *p* and we observe that it seems to converge at the same rate to 0.5 than in the previous case.

From these illustrations, we also conclude that the average Age of Information is not monotone with respect to α (note that in [19] it has been shown that the average Age of Information of a system with a single server with energy harvesting is monotone with respect to α). For instance, we see that, when p=0.45 and λ=10, the average Age of Information increases when λ is small, then decreases and finally decreases again.

### 4.3. Analysis of ther Parameter p

**Figure 8:**
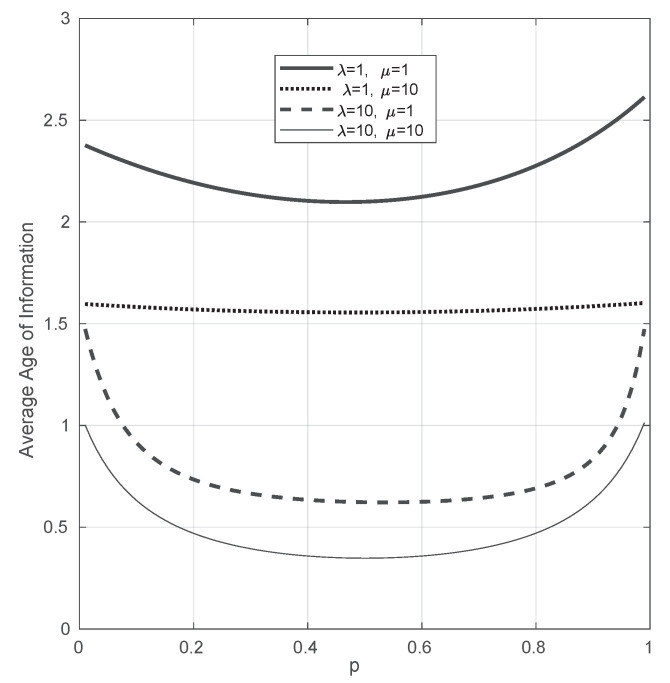
Average Age of Information with respect to *p* for different values of λ and μ, when *p* varies from 0.01 to 0.99. α=1.

We now focus on the parameter *p* that determines the proportion of the total incoming arrival rate is sent to each of the servers. In Figure 8, we consider α=1 and we plot the average Age of Information when *p* varies from 0.01 to 0.99 for different values of λ and μ. We observe that, in all the considered instances, the average Age of Information first decreases with *p* and then increases. This suggests that the minimum of the average Age of Information for *p* is achieved when this parameter is close to 0.5, i.e., in the symmetric case that has been studied in Section 4.1.

## 5. Conclusions

We consider a system with two transmission channels that do not communicate and a energy source that generates energy following a random process. We model this system as a system with Poisson arrivals of status updates to two parallel servers and of energy packets to a battery. We study the average Age of Information of system using the Stochastic Hybrid System method. We first show that the average Age of Information of this system can be computed by solving a system of 8 linear equations (Proposition 2). We then consider that the arrival rate tends to infinity and, in this case, we show that the average Age of Information is equal to one divided by the sum of the service rate of the servers. This implies that, in this regime, the average Age of Information does not depend on the probability to dispatch jobs to the server and on the arrival rate of energy packets. We then study numerically the performance of our model with single server systems with energy harvesting and the same load of data packets as in our model. We conclude that, when the arrival rate of energy packets tends to infinity, the same conclusions of [18] follow in our model (i.e., the average Age of Information does not satisfy the same properties that other performance measures used in queuing theory such as number of packets in the queue). However, when the arrival rate of energy packets is not large, we conclude that the parallel server system outperforms the single server systems.

For future work, we would like to analyze the average Age of Information with a larger number of servers and with buffer for the energy packets and the data packets. Furthermore, we would like to study optimality of this model for some parameters such as *p*. We would also like to extend this model to include other parameters of the system such as transmit power or channel statistics to address real-life problems. Finally, we are also interested in exploring the performance of this model when it requires energy not only to send a status update to the monitor after getting service, but also to receive data packets from the source.

## Data Availability

The code to reproduce the results of this article are available at https://github.com/josudoncel/AioParallelEnergy, accessed on 18 November 2021.

## Appendix A. Average Age of Information and SHS

In the SHS, the system is modeled as a hybrid state (q(t),x(t)), where q(t) a state of a continuous time Markov Chain and x(t) is a vector whose components is the generation time of each of the updates. We assume that in the monitor there is the update with the latest generation time.

A link *l* of the Markov Chain represents a transition between two states, which occurs with rate $\lambda^{l}$. In each transition *l*, the vector x changes to $x^{\prime }$ using a transformation matrix $A_{l}$, that is, $x^{\prime }=xA_{l}$. Therefore, x(t) is a piece-wise function.

For each state *q* of the Markov Chain, we define $b_{q}$ as the vector whose elements are zero or one. One values represent the packets that are present in the system and therefore that the time from their generation increases at unit rate. On the other hand, zero values represent the updates that are not in the system.

We assume the Markov Chain is ergodic and we denote by $\pi_{q}$ the stationary distribution of state *q*. We denote by $L_{q}$ the set of outgoing links of state *q* and $L_{q}^{\prime }$ the set of links that get into state *q*. We now present the following theorem that will be used to characterize the average Age of Information:

**Theorem** **A1** ([17], Thm 4). *Let* $v_{q}\left(i\right)$ *denote the i-th element of the vector* $v_{q}$. For each state q, if $v_{q}$ *is a non-negative solution of the following system of equations*

(A1) $$v_{q}\sum_{l\in L_{q}}\lambda^{l}=b_{q}\pi_{q}+\sum_{l\in L_{q}^{\prime }}\lambda^{l}v_{q_{l}}A_{l},$$

*then the average Age of Information is* $\Delta =\sum_{q}v_{q}\left(0\right)$*.*

## Appendix B. Proof of Proposition 1

We use the SHS technique to compute the average age of information of this system. We denote by $x=[x_{0}\left(t\right)\;x_{1}\left(t\right)\;x_{2}\left(t\right)]$, where $x_{0}\left(t\right)$ represents the age of the information at time t and $x_{i}\left(t\right)$ the age of information if a data packet that is getting service in data queue *i* is sent successfully to the monitor, i=1,2.

The Markov Chain of this model is presented in Figure A1. We denote by ijk the state where there are *i* data packets in data queue 1, *j* data packets in data queue 2 and *k* energy packets in the energy queue. We now describe each of the transitions of this model.

l=0 A data packet arrives to data queue 1 when the system is empty. Therefore, the data packet starts getting service and the value of $x_{1}$ is set to zero, that is, $x_{1}^{\prime }=0$. The rest of values of x are not modified.
l=1 A data packet of data queue 1 ends service when data queue 2 is empty. The data packet is sent to the energy queue, which is empty. Hence, the data packet is lost and the system gets empty. The vector x is not modified.
l=2 A data packet arrives to data queue 1 when it is full. For this case, the last arrived packet replaces the packet in service and the age of $x_{1}$ changes to zero since the last arrived packet is fresh, that is, $x_{1}^{\prime }=0$. The rest of values of x do not change.
l=3 A data packet arrives to data queue 1 when the energy queue is full. Therefore, the data packet starts getting service and the value of $x_{1}$ is set to zero, that is, $x_{1}^{\prime }=0$. The rest of values of x are not modified.
l=4 An energy packet arrives to the system when data queue 1 is full and data queue 2 is empty. The arrival of an energy packet does not modify the age of the packets in the system and of the monitor, therefore the vector x is not modified.
l=5 A data packet is data queue 1 ends service and it is sent to the energy queue, which contains one energy packet. Therefore, the packet is successfully delivered and the age of information is modified by the age of the served packet, that is, $x_{0}^{\prime }=x_{1}$.
l=6 A data packet arrives to data queue 2 when the data queue 1 is full and the data queue 2 and the energy queue were empty. For this case, the value of $x_{2}$ is set to zero, that is, $x_{2}^{\prime }=0$ and the rest of the values of x are not modified.
l=7 A data packet in data queue 2 ends service when the energy queue is empty and data queue 1 is full. Therefore, the served packet is sent to the energy queue and it is lost. The vector x is not modified.
l=8 A data packet in data queue 2 ends service and the system is full. Therefore, the served packet is delivered and thus, the value of $x_{0}$ is replaced by that of $x_{2}$, that is, $x_{0}^{\prime }=x_{2}$. The rest of the values of x are not modified.
l=9 A data packet arrives to data queue 2 when the energy queue and data queue 1 are full. Therefore, all the queues are full and the value of $x_{2}$ is set to zero since a fresh packet arrived to that queue, that is, $x_{2}^{\prime }=0$. The rest of the values of x are not modified.
l=10 A data packet arrives to data queue 1 or there is an energy packet arrival when the energy queue and data queue 1 are full and data queue 2 is empty. In the former case, the value of $x_{1}$ changes to zero since a fresh packet arrived to that queue, that is, $x_{1}^{\prime }=0$ and the rest of the values of x are not modified, whereas in the later case the vector x does not change.

With the roles of data queue 1 and data queue 2 reversed, transitions 11–21 coincide with 0–10, respectively. Besides, transitions 21–24 represent an arrival of an energy queue, which increases by one the number of energy packets if the energy queue is empty, but the vector x is never modified. Finally, we focus on transitions 25 and 26:

l=25 The data queues are full the energy queue is empty. If a data packet arrives to data queue 1, the value of $x_{1}$ changes to zero since a fresh packet arrived to that queue, that is, $x_{1}^{\prime }=0$ and the rest of the values of x are not modified. Likewise, if a data packet arrives to data queue 2, the value of $x_{2}$ changes to zero since a fresh packet arrived to that queue, that is, $x_{2}^{\prime }=0$ and the rest of the values of x are not modified.
l=26 The system is full and one of the following events occur: (i) a data packet arrives to data queue 1, (ii) a data packet arrives to data queue 2 and (iii) an energy packet arrives to the energy queue. For the case (i), (resp. the case (ii)) the value of $x_{1}$ changes to zero (resp. $x_{2}$ changes to zero) since a fresh packet arrived to that queue, that is, $x_{1}^{\prime }=0$ (resp. $x_{2}^{\prime }=0$) and the rest of the values of x are not modified. Finally, for the case (iii), the vector x is not modified since an arrival of an energy queue does not change the age of the packets in the system.

**Table A1:** Table of SHS transitions of Figure A1.

l	$q_{l}\to q_{l^{\prime }}$	$\lambda^{l}$	$x^{\prime }=xA_{l}$	${\bar{v}}_{q_{l}}A_{l}$
0	000→100	λp	$\left[x_{0}\;0\;0\right]$	$\left[v_{000}\left(0\right)\;0\;0\right]$
1	100→000	μ	$\left[x_{0}\;0\;0\right]$	$\left[v_{100}\left(0\right)\;0\;0\right]$
2	100→100	λp	$\left[x_{0}\;0\;0\right]$	$\left[v_{100}\left(0\right)\;0\;0\right]$
3	001→101	λp	$\left[x_{0}\;0\;0\right]$	$\left[v_{001}\left(0\right)\;0\;0\right]$
4	100→101	α	$\left[x_{0}\;0\;0\right]$	$\left[v_{100}\left(0\right)\;0\;0\right]$
5	101→000	μ	$\left[x_{1}\;0\;0\right]$	$\left[v_{101}\left(1\right)\;0\;0\right]$
6	100→110	λ(1−p)	$\left[x_{0}\;x_{1}\;0\right]$	$\left[v_{100}\left(0\right)\;v_{100}\left(1\right)\;0\right]$
7	110→100	μ	$\left[x_{0}\;x_{1}\;0\right]$	$\left[v_{110}\left(0\right)\;v_{110}\left(1\right)\;0\right]$
8	111→100	μ	$\left[x_{2}\;x_{1}\;0\right]$	$\left[v_{111}\left(2\right)\;v_{111}\left(1\right)\;0\right]$
9	101→111	λ(1−p)	$\left[x_{0}\;x_{1}\;0\right]$	$\left[v_{101}\left(0\right)\;v_{101}\left(1\right)\;0\right]$
10	101→101	λp	$\left[x_{0}\;0\;0\right]$	$\left[v_{101}\left(0\right)\;0\;0\right]$
		α	$\left[x_{0}\;x_{1}\;0\right]$	$\left[v_{101}\left(0\right)\;v_{101}\left(1\right)\;0\right]$
11	000→010	λ(1−p)	$\left[x_{0}\;0\;0\right]$	$\left[v_{000}\left(0\right)\;0\;0\right]$
12	010→000	μ	$\left[x_{0}\;0\;0\right]$	$\left[v_{010}\left(0\right)\;0\;0\right]$
13	010→010	λ(1−p)	$\left[x_{0}\;0\;0\right]$	$\left[v_{010}\left(0\right)\;0\;0\right]$
14	001→011	λ(1−p)	$\left[x_{0}\;0\;0\right]$	$\left[v_{001}\left(0\right)\;0\;0\right]$
15	010→011	α	$\left[x_{0}\;0\;x_{2}\right]$	$\left[v_{010}\left(0\right)\;0\;v_{010}\left(2\right)\right]$
16	011→000	μ	$\left[x_{2}\;0\;0\right]$	$\left[v_{011}\left(2\right)\;0\;0\right]$
17	010→110	λp	$\left[x_{0}\;0\;x_{2}\right]$	$\left[v_{010}\left(0\right)\;0\;v_{010}\left(2\right)\right]$
18	110→010	μ	$\left[x_{0}\;0\;x_{2}\right]$	$\left[v_{110}\left(0\right)\;0\;v_{110}\left(2\right)\right]$
19	111→010	μ	$\left[x_{1}\;0\;x_{2}\right]$	$\left[v_{111}\left(1\right)\;0\;v_{111}\left(2\right)\right]$
20	011→111	λp	$\left[x_{0}\;0\;x_{2}\right]$	$\left[v_{011}\left(0\right)\;0\;v_{011}\left(2\right)\right]$
21	011→011	λ(1−p)	$\left[x_{0}\;0\;0\right]$	$\left[v_{011}\left(0\right)\;0\;0\right]$
		α	$\left[x_{0}\;0\;x_{2}\right]$	$\left[v_{011}\left(0\right)\;0\;v_{011}\left(2\right)\right]$
22	000→001	α	$\left[x_{0}\;0\;0\right]$	$\left[v_{000}\left(0\right)\;0\;0\right]$
23	001→001	α	$\left[x_{0}\;0\;0\right]$	$\left[v_{001}\left(0\right)\;0\;0\right]$
24	110→111	α	$\left[x_{0}\;x_{1}\;x_{2}\right]$	$\left[v_{110}\left(0\right)\;v_{110}\left(1\right)\;v_{110}\left(2\right)\right]$
25	110→110	λp	$\left[x_{0}\;0\;x_{2}\right]$	$\left[v_{110}\left(0\right)\;0\;v_{110}\left(2\right)\right]$
		λ(1−p)	$\left[x_{0}\;x_{1}\;0\right]$	$\left[v_{110}\left(0\right)\;v_{110}\left(1\right)\;0\right]$
26	111→111	λp	$\left[x_{0}\;0\;x_{2}\right]$	$\left[v_{111}\left(0\right)\;0\;v_{111}\left(2\right)\right]$
		λ(1−p)	$\left[x_{0}\;x_{1}\;0\right]$	$\left[v_{111}\left(0\right)\;v_{111}\left(1\right)\;0\right]$
		α	$\left[x_{0}\;x_{1}\;x_{2}\right]$	$\left[v_{111}\left(0\right)\;v_{111}\left(1\right)\;v_{111}\left(2\right)\right]$

We represent the evolution of x for all the above transitions in Table A1. We now that the information is presented in the same way as in Table A1, that is, in each row, each transition is represented in a different row, in the second column of the table, the origin and the destination state of the Markov Chain, in the third column the rate of each transition, whereas in the last two columns we show the evolution of x and ${\bar{v}}_{q_{l}}A_{l}$, respectively.

Let $Q^{p}$ be the set of state of the Markov Chain of Figure A1. The stationary distribution of state $q\in Q^{p}$ is denoted by $\pi_{q}$. We now focus on the stationary distribution of the Markov Chain of Figure A1. The balance equations are provided next:

$$\begin{array}{cc} \pi_{000}\left(\lambda +\alpha \right) & =\pi_{101}\mu +\pi_{100}\mu +\pi_{011}\mu +\pi_{010}\mu \\ \pi_{001}\lambda & =\pi_{000}\alpha \\ \pi_{010}\left(\lambda p+\mu +\alpha \right) & =\pi_{000}\lambda \left(1-p\right)+\pi_{110}\mu +\pi_{111}\mu \\ \pi_{011}\left(\lambda p+\mu \right) & =\pi_{010}\alpha +\pi_{001}\lambda \left(1-p\right)\\ \pi_{100}\left(\lambda \left(1-p\right)+\alpha +\mu \right) & =\pi_{000}\lambda p+\pi_{110}\mu +\pi_{111}\mu \\ \pi_{101}\left(\lambda \left(1-p\right)+\mu \right) & =\pi_{001}\lambda p+\pi_{100}\alpha \\ \pi_{110}\left(\alpha +2\mu \right) & =\pi_{010}\lambda p+\pi_{100}\lambda \left(1-p\right)\\ \pi_{111}2\mu & =\pi_{011}\lambda p+\pi_{101}\lambda \left(1-p\right)+\pi_{110}\alpha \end{array}$$

The Markov chain under study is clearly ergodic. Therefore, there always exists a unique solution of the above system of equations that satisfies $\sum_{q\in Q^{p}}\pi_{q}=1$.

We remark that the above system of equations coincides with Equation (1a–h).

We now define the vector $b_{q}$ for any $q\in Q^{p}$. For q∈{000,001}, we have that $b_{q}=\left[1\;0\;0\right]$, whereas for q∈{100,101}, $b_{q}=\left[1\;1\;0\right]$, for q∈{010,011}, $b_{q}=\left[1\;0\;1\right]$ and for q∈{110,111}, $b_{q}=\left[1\;1\;1\right]$. Besides, for all $q\in Q^{p}$, we denote by $v_{q}\left(i\right)$ the *i*-th component of vector $v_{q}$, with i=0,1,2.

We now aim to apply Theorem A1 to this model and, from (A1), it follows that

(A2a) $$\begin{array}{cc} v_{000}\left(\lambda +\alpha \right)= & \left[\pi_{000}\;0\;0\right]+\mu \left[v_{100}\left(0\right)\;0\;0\right]\\ & +\mu \left[v_{101}\left(1\right)\;0\;0\right]+\mu \left[v_{010}\left(0\right)\;0\;0\right]\\ & +\mu [v_{011}\left(2\right)\;0\;0] \end{array}$$

(A2b) $$\begin{array}{cc} v_{001}\left(\lambda +\alpha \right)= & \left[\pi_{001}\;0\;0\right]+\alpha \left[v_{000}\left(0\right)\;0\;0\right]\\ & +\alpha [v_{001}\left(0\right)\;0\;0] \end{array}$$

(A2c) $$\begin{array}{cc} v_{010}\left(\lambda +\alpha +\mu \right)= & \left[\pi_{010}\;0\;\pi_{010}\right]+\lambda \left(1-p\right)\left[v_{000}\left(0\right)\;0\;0\right]\\ & +\lambda \left(1-p\right)[v_{010}\left(0\right)\;0\;0]\\ & +\mu [v_{111}\left(1\right)\;0\;v_{111}\left(2\right)] \end{array}$$

(A2d) $$\begin{array}{cc} v_{011}\left(\lambda +\alpha +\mu \right)= & \left[\pi_{011}\;0\;\pi_{011}\right]+\alpha \left[v_{010}\left(0\right)\;0\;v_{010}\left(2\right)\right]\\ & +\alpha [v_{011}\left(0\right)\;0\;v_{011}\left(2\right)]\\ & +\lambda \left(1-p\right)[v_{001}\left(0\right)\;0\;0]\\ & +\lambda \left(1-p\right)[v_{011}\left(0\right)\;0\;0] \end{array}$$

(A2e) $$\begin{array}{cc} v_{100}\left(\lambda +\alpha +\mu \right)= & \left[\pi_{100}\;\pi_{100}\;0\right]+\lambda p\left[v_{000}\left(0\right)\;0\;0\right]\\ & +\lambda p[v_{100}\left(0\right)\;0\;0]\\ & +\mu [v_{111}\left(2\right)\;v_{111}\left(1\right)\;0] \end{array}$$

(A2f) $$\begin{array}{cc} v_{101}\left(\lambda +\alpha +\mu \right)= & \left[\pi_{101}\;\pi_{101}\;0\right]+\lambda p\left[v_{001}\left(0\right)\;0\;0\right]\\ & +\lambda p[v_{101}\left(0\right)\;0\;0]\\ & +\alpha [v_{101}\left(0\right)\;v_{101}\left(1\right)\;0]\\ & +\alpha [v_{100}\left(0\right)\;v_{100}\left(1\right)\;0] \end{array}$$

(A2g) $$\begin{array}{cc} v_{110}\left(\lambda +2\mu +\alpha \right)= & \left[\pi_{110}\;\pi_{110}\;\pi_{110}\right]\\ & +\lambda p[v_{010}\left(0\right)\;0\;v_{010}\left(2\right)]\\ & +\lambda p[v_{110}\left(0\right)\;0\;v_{110}\left(2\right)]\\ & +\lambda \left(1-p\right)[v_{100}\left(0\right)\;v_{100}\left(1\right)\;0]\\ & +\lambda \left(1-p\right)[v_{110}\left(0\right)\;v_{110}\left(1\right)\;0] \end{array}$$

(A2h) $$\begin{array}{cc} v_{111}\left(\lambda +2\mu \alpha \right)= & \left[\pi_{111}\;\pi_{111}\;\pi_{111}\right]\\ & +\lambda p[v_{011}\left(0\right)\;0\;v_{011}\left(2\right)]\\ & +\lambda p[v_{111}\left(0\right)\;0\;v_{111}\left(2\right)]\\ & +\lambda \left(1-p\right)[v_{101}\left(0\right)\;v_{101}\left(1\right)\;0]\\ & +\lambda \left(1-p\right)[v_{111}\left(0\right)\;v_{111}\left(1\right)\;0]\\ & +\alpha [v_{110}\left(0\right)\;v_{110}\left(1\right)\;v_{110}\left(2\right)]\\ & +\alpha [v_{111}\left(0\right)\;v_{111}\left(1\right)\;v_{111}\left(2\right)]. \end{array}$$

We note that the above expression is the same as Equation (2a–p) with the following change of variable: $v_{000}\left(0\right)=x_{1},v_{001}\left(0\right)=x_{2},\ldots ,v_{111}\left(1\right)=x_{15},v_{111}\left(2\right)=x_{16}$ and $s_{0}=\pi_{000},$$s_{1}=\pi_{001},\ldots ,s_{7}=\pi_{111}$. Using Theorem A1, the desired result follows.

## Appendix C. Proof of Lemma 1

We first write the Equation (2d,f,m,p), :

(A3a) $$\begin{array}{cc} -s_{2}= & -x_{4}\left(\lambda +\alpha +\mu \right)+\mu x_{16} \end{array}$$

(A3b) $$\begin{array}{cc} -s_{3}= & -x_{6}\left(\lambda +\mu \right)+\alpha x_{4} \end{array}$$

(A3c) $$\begin{array}{cc} -s_{6}= & -x_{13}\left(\lambda \left(1-p\right)+2\mu +\alpha \right)+\lambda px_{4} \end{array}$$

(A3d) $$\begin{array}{cc} -s_{7}= & -x_{16}\left(\lambda \left(1-p\right)+2\mu \right)+\lambda px_{6}+\alpha x_{13}, \end{array}$$

And we observe that it is a system with 4 linear equations with 4 variables. Moreover, from (A3b), we get

$$x_{6}=\frac{\alpha x_{4}+s_{3}}{\lambda +\mu },$$

which gives (6), whereas from (A3c) we get

$$x_{13}=\frac{\lambda px_{4}+s_{6}}{\lambda (1-p)+2\mu +\alpha }.$$

Substituting these expression in Equation (A3a–d), we obtain the following system of equations:

(A4a) $$\begin{array}{cc} -s_{2}= & -x_{4}\left(\lambda +\alpha +\mu \right)+\mu x_{16}\\ -s_{7}= & -x_{16}\left(\lambda \left(1-p\right)+2\mu \right)+\lambda p\frac{\alpha x_{4}+s_{3}}{\lambda +\mu } \end{array}$$

(A4b) $$\begin{array}{c} +\alpha \frac{\lambda px_{4}+s_{6}}{\lambda (1-p)+2\mu +\alpha }, \end{array}$$

From (A4a), it results that

$$x_{4}\left(\lambda +\alpha +\mu \right)=\mu x_{16}+s_{2}\Leftrightarrow x_{4}=\frac{\mu x_{16}+s_{2}}{\lambda +\alpha +\mu },$$

which is equal to (5) and substituting the obtained expression in (A4b) and simplifying, we get (4). And the desired result follows.

## Appendix D. Proof of Lemma 3

We first note that, from (1a), we have that

$$s_{0}=\frac{s_{2}\mu +s_{3}\mu +s_{4}\mu +s_{5}\mu }{\lambda +\alpha },$$

which tends to zero when λ→∞ because $s_{i}<1$ for all *i* and μ<∞. From (1b), we get that

$$s_{1}=\frac{s_{0}\alpha }{\lambda },$$

which, using the previous result and α<∞, also proves that $s_{1}$ tends to zero when λ→∞ because $s_{0}<1$. We now focus on (1c),:

$$s_{2}=\frac{s_{0}\lambda \left(1-p\right)+s_{6}\mu +s_{7}\mu }{\lambda p+\mu +\alpha },$$

and when λ→∞, we have that $s_{2}$ tends to $s_{0}\left(1-p\right)/p$ and this tends to zero because $s_{0}$ tends to zero. We now study (1d) and, using that $\alpha s_{0}=\lambda s_{1}$ (see (1b)), we get

$$s_{3}=\frac{s_{0}\alpha \left(1-p\right)+s_{2}\alpha }{\lambda p+\mu },$$

F which also tends to zero when λ→∞ because $s_{0}<1$ and α<∞. From (1e), we obtain

$$s_{4}=\frac{s_{0}\lambda p+s_{6}\mu +s_{7}\mu }{\lambda (1-p)+\alpha +\mu },$$

and we observe that $s_{4}$ tends to $s_{0}p/\left(1-p\right)$ when λ→∞ and, since $s_{0}$ tends to zero, so does $s_{4}$. Finally, we have from (1f) and using that $\alpha s_{0}=\lambda s_{1}$ (see (1b)),

$$s_{5}=\frac{s_{0}\alpha p+s_{4}\alpha }{\lambda (1-p)+\mu },$$

which also tends to zero when λ→∞. And the desired result follows.
